# Shape and Displacement Fluctuations in Soft Vesicles Filled by Active Particles

**DOI:** 10.1038/srep34146

**Published:** 2016-09-28

**Authors:** Matteo Paoluzzi, Roberto Di Leonardo, M. Cristina Marchetti, Luca Angelani

**Affiliations:** 1 Department of Physics and Syracuse Soft Matter Program, Syracuse University, Syracuse NY 13244, USA; 2 Dipartimento di Fisica Università Sapienza, P.le A Moro 2, 00185 Rome, Italy; 3 NANOTEC-CNR, Institute of Nanotechnology, Soft and Living Matter Laboratory, Piazzale A. Moro 2, I-00185, Roma, Italy; 4 ISC-CNR, Institute for Complex Systems, Piazzale A. Moro 2, I-00185 Roma, Italy

## Abstract

We investigate numerically the dynamics of shape and displacement fluctuations of two-dimensional flexible vesicles filled with active particles. At low concentration most of the active particles accumulate at the boundary of the vesicle where positive particle number fluctuations are amplified by trapping, leading to the formation of pinched spots of high density, curvature and pressure. At high concentration the active particles cover the vesicle boundary almost uniformly, resulting in fairly homogeneous pressure and curvature, and nearly circular vesicle shape. The change between polarized and spherical shapes is driven by the number of active particles. The center-of-mass of the vesicle performs a persistent random walk with a long time diffusivity that is strongly enhanced for elongated active particles due to orientational correlations in their direction of propulsive motion. In our model shape-shifting induces directional sensing and the cell spontaneously migrate along the polarization direction.

Active systems are collections of agents that convert the energy of the environment in systematic movement^1^,^2^,^3^. Examples include bacterial colonies^4^, epithelial cell layers^5^, self-propelled colloids^6^, swimming microorganisms^7^, schools of fish^8^ and bird flocks^9^. Active particles can form gas, liquid, liquid crystal or glassy phases with structural properties remarkably similar to those of ordinary materials^10^,^11^,^12^,^13^,^14^,^15^,^16^. Active systems are, however, out-of-equilibrium. Hence their steady state is not described by the Boltzmann distribution and they can support spontaneous, self-sustained motion, which can in turn be enhanced, stabilized or suppressed by suitably designed confining geometries^17^,^18^,^19^,^20^. It has been shown that active agents can give rise to ratchet effects^21^,^22^,^23^,^24^, power microgears^25^,^26^,^27^, drive spontaneous accumulations of passive colloids over target regions^28^, and exhibit long lived density fluctuations^29^. From a theoretical point of view, the effect of confinement has been used to investigate the concept of pressure in active systems^30^,^31^,^32^,^33^ and the effect of wall curvature on both active particles^34^ and passive tracers^35^. Strong confinement can induce hysteretic dynamics^36^ or sustained spontaneous density oscillations^37^. The role of curved walls on active gas has been investigated in ref. 38.

Previous work has focused on confinement by rigid walls. While recent studies have investigated the effects of active baths on flexible open chains^39^,^40^,^41^, the case of swimmers confined by deformable boundaries has recently been analyzed only for case of spherical active Brownian particles by Tian *et al*.^42^. An interesting example of active colloidal cell driven by micro rotators has been theoretically investigated in ref. 43. Here we consider an active vesicle in two dimensions composed by a flexible one dimensional membrane enclosing active particles representing an active solute. The corresponding equilibrium system would be a vesicle filled with a suspension and bounded by a flexible membrane that is permeable to the solvent but not to the solute molecules. In this case, the solute concentration would be uniform throughout the vesicle interior and exert a homogeneous pressure on the membrane whose equilibrium configuration would be spherical, or circular in two dimensions. When the solute molecules are active particles or microswimmers, we find that only for high densities of active particles the membrane shape fluctuates around a circle. When the swimmers packing fraction falls below a characteristic value, depending on particles shape, the vesicle acquires an asymmetric shape characterized by a bimodal distribution of the local curvatures, with a high curvature peak and a near zero curvature component. This effect is driven by a feedback mechanism coupling swimmers density and membrane curvature through local pressure. A local fluctuation of particle density produces a local pressure increase that induces a larger curvature on the flexible membrane. Since active particles tend to accumulate at concave boundaries, this local curvature increase drives further accumulation of swimmers, which in turn raises the local pressure. The presence of this feedback mechanism is confirmed by a strong correlation between the local swimmers density (or local pressure on the membrane) and the local curvature of the membrane. Finally, we examine the center of mass dynamics of the whole vesicle and show that it performs a persistent random walk with a long time diffusivity that is larger for elongated swimmers due to orientational correlations. Interesting, the resulting migratory behavior shares some similarities with Eukaryotic directed cell migration^44^,^45^.

## Methods

We perform two dimensional simulations of *N*_*s*_ *run-and-tumble* swimmers of width *a* and length 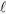 (aspect ratio 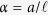) confined by a deformable membrane. We specifically consider swimmers of two different aspect ratios, *α* = 1/2 (elongated) and *α* = 1 (spherical).

### Swimmers

We consider *N*_*s*_ run-and-tumble particles in two dimensions. The model is the same used in refs 25, 46, 47, 48, 49 to describe *E. coli* bacterial suspensions. Each particle consists of a chain of *p* rigidly connected disks of diameter *a* aligned along the swimming direction 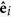. We denote by ***r***_*i*_ the center of mass of the *i*th swimmer. The position 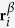, with *β* = 1, …, *p*, of the *β*-th disk on the *i*-th swimmer is then


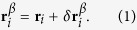


**Figure 1:**
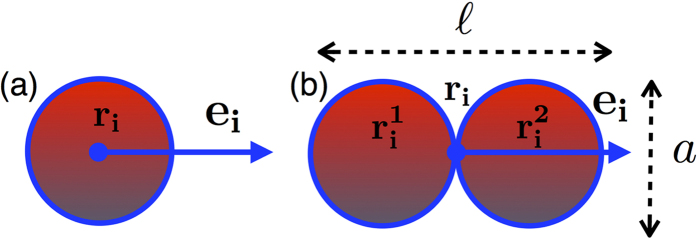
Pictorial representation of the swimmers. Each swimmer consists of *p* spherically symmetric force centers aligned along the swimming direction **e**_*i*_, with *p* = 1 describing spherical particles (panel a) and *p* = 2 elongated ones (panel b).

Here we consider *p* = 1, corresponding to spherical swimmers with 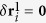, and *p* = 2, corresponding to elongated swimmers, with 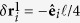 and 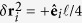 (panel (b) in Fig. 1). We assume swimmers interact only through steric repulsion and that the interaction potential is written as the sum of radially symmetric potentials centered at each disk. For this reasons the individual disks that compose our swimmers are also referred to as force centers. At low Reynolds number, the equations of motion of the *i*-th swimmer are


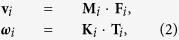


where **v**_*i*_ is the center of the mass velocity and ***ω***_*i*_ the angular velocity of the *i*-th swimmer. **M**_*i*_ and **K**_*i*_ are the translational and rotational mobility matrices


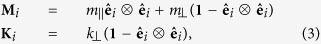


the symbol ⊗ is the dyadic product and **1** the identity matrix. In Eq. (2), **F**_*i*_ and **T**_*i*_ are the total force and the total torque acting on the of the *i*-th swimmer, given by


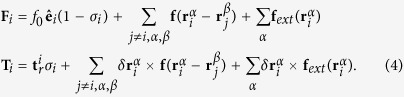


The index *j* = 1, …, *N*_*s*_ runs over swimmers, the indices *α* = 1, .., *p* and *β* = 1, ..., *p* run over disks, and *σ*_*i*_ is a state variable, with value 0 for running swimmers and 1 for tumbling ones. During the running state each swimmer is self-propelled along 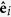 with self-propulsion speed 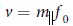. In the tumbling state, the random torque 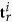 rotates the swimming direction 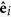 at the tumbling rate, *λ*. Moreover, it takes a finite time (*λ*10)^−1^ for the swimmers to reorient the swimming direction. The external force 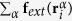 in Eq. (4) represent the interaction of the swimmers with the flexible confining boundary. The details of this interaction will be specified in the next section. Finally, the repulsive force **f**(**r**) is conservative and generated by the potential 
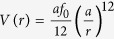
25. Below we choose units such that 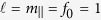 and use *λ* = 0.1, *k*_⊥_ = 4.8 and *m*_⊥_ = 0.87.

### Membrane

The bounding membrane is modeled as a ring of *N*_*b*_ colloidal beads of diameter *a* connected by springs. Denoting with **R**_*n*_ the position of *n*-th bead, the equation of motion of the membrane in the low Reynolds number regime is given by


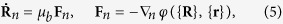


where the potential *φ*({**R**}, {**r**}) consists of harmonic and repulsive parts, *φ*({**R**}, {**r**}) = *φ*({**R**})^*harm*^ + *φ*({**R**}, {**r**})^*rep*^, with


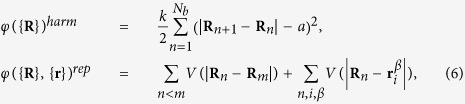


where 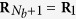 in the sum in Eq. (6). We choose *μ*_*b*_ = *μ*, *k* = 5 · 10^2^. The external force in Eq. (4) is 
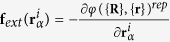
.

The initial configuration of the membrane is a circle of radius *R*_0_ = *a*(2sin(*π*/*N*_*b*_))^−1^ and area 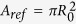. The swimmers cover a fraction *ϕ* = *N*_*s*_*a*_*swim*_/*A*_*ref*_ of the initial area of the vescicle, with *a*_*swim*_ = *pπ*(*a*/2)^2^ the area of one swimmer. The entire vesicle moves in a two dimensional box of side 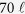 with periodic boundary conditions. We have performed numerical simulations of membranes composed of *N*_*b*_ = 50, 100, 150 beads enclosing *N*_*s*_ elongated swimmers (*p* = 2) with packing fraction from *ϕ* = 0.07 up to 0.83 and *N*_*s*_ spherical swimmer (*p* = 1) with packing fraction from *ϕ* = 0.05 to 0.82. Specifically, in the case of elongated swimmers we have simulated systems with *N*_*s*_ = 12, 21, 32, 37, 52, 69, 80 for *N*_*b*_ = 50, *N*_*s*_ = 52, 69, 80, 97, 112, 137, 156, 208, 225, 256, 316, 384, 421, 448 for *N*_*b*_ = 100, and *N*_*s*_ = 80, 112, 156, 208, 256, 316, 384, 448, 540, 616, 716, 812, 973 for *N*_*b*_ = 150. For spherical swimmers we have used *N*_*s*_ = 12, 21, 32, 52, 80, 112, 156, 208 for *N*_*b*_ = 50, *N*_*s*_ = 52, 112, 208, 316, 448, 616, 812 for *N*_*b*_ = 100, and *N*_*s*_ = 316, 448, 616, 812, 1020, 1264 for *N*_*b*_ = 150.

To quantify the shape of the membrane we measure the gyration tensor ***Q***, given by


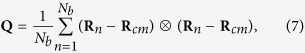


with **R**_*cm*_ the center of the mass of membrane beads. From the average values of the two eigenvalues *λ*_1_ and *λ*_2_ of **Q** we compute the squared radius of gyration 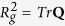 that gives a measure of the extension of the cell,


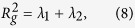


and the asphericity^50^


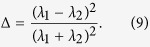


The value Δ = 0 corresponds to a circle and Δ = 1 to a rod. Since the gyration tensor is a dynamical quantity, the observables *R*_*g*_ and Δ are computed from the time average of the eigenvalues.

We characterize the local shape of the membrane by measuring the local curvature, *κ*, defined as^51^


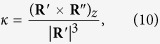


where **R** is the vector position of a membrane point, **R**′ and **R**^′′^ are the first and second derivatives of **R** with respect to the membrane contour length. Curvature values are evaluated at the beads position along the membrane, using discrete form of the derivatives. To evaluate the pressure *P* on the *n*-th bead, we have computed numerically the total force that swimmers exert along the local normal 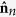 to the membrane divided by the average length of the segments connecting such a bead to its neighbors.

## Results

**Figure 2:**
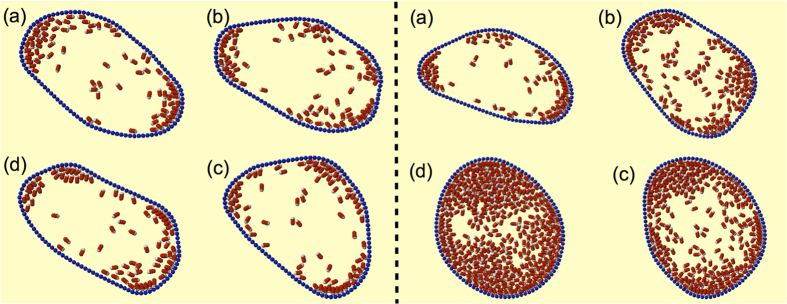
Shape fluctuations. The bounding membrane is composed of *N*_*b*_ = 100 beads. Left panel: snapshots of vesicle shapes explored by the active vesicle for low packing fraction of elongated swimmers (*ϕ* = 0.16). Right panel: the vesicle becomes more symmetric as the number of active particles increases, *ϕ* is 0.16 (**a**), 0.31 (**b**), 0.51 (**c**) and 0.76 (**d**).

It is well established in the literature that confined active particles tend to accumulate along the confining walls^15^,^32^,^52^. In our case the confining walls are flexible and swimmer accumulation induces strong distortions of the bounding membrane. These distortions are evident in the snapshots shown in Fig. 2 where elongated swimmers are bounded by a membrane of *N*_*b*_ = 100 beads. For low packing fraction (left panel) the membrane explores different shapes characterized by regions of high curvature. As the number of swimmers is increased (right panel), the imbalance of particles along the flexible walls becomes less dramatic and the vesicle assumes more symmetric shapes.

### Pressure and global shape properties

We first discuss the case of spherical swimmers (*p* = 1, aspect ratio *α* = 1). In this case particle reorientations are solely due to tumbles and no aligning interactions exists between swimmers or swimmers and walls.

**Figure 3:**
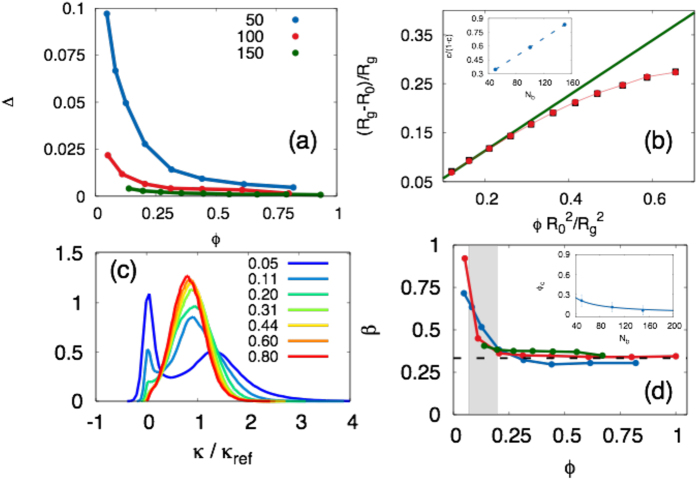
Membrane shape for spherical swimmers. (**a**) Asphericity parameter for *N*_*b*_ = 50 (blue symbols), *N*_*b*_ = 100 (red symbols), and *N*_*b*_ = 150 (green symbols), the lines are a guide to the eye. The membrane approaches a circular shape with increasing *ϕ*. (**b**) The red circles are (*R*_*g*_ − *R*_0_)/*R*_*g*_ (the red line is a guide to the eye), the black symbols represent *PN*_*b*_/2*πk*, and the green line is the fit to Eq. (15). The data are plotted as a function of the area fraction computed with respect the circle of radius *R*_*g*_ for *N*_*b*_ = 150. Inset: the quantity *c*/(1−*c*) as a function of *N*_*b*_. (**c**) Probability distributions of the local curvatures for *N*_*b*_ = 100 for increasing *ϕ* from 0.05 (blue) to 0.80 (red). (**d**) Parameter *β* as a function of *ϕ* for *N*_*b*_ = 50, 100, 150 (blue, red and green), the black dashed line is *β* for a Gaussian distribution. The grey area represents the estimated *ϕ*_*c*_ range. Inset: *ϕ*_*c*_ obtained from the decoupling between pressure and deformation (blue symbols) the line is the estimate of *ϕ*_*c*_ given by *ϕ* (*N*_*b*_ = *N*_*s*_).

To quantify the deviations of the active vesicle from circular shape we display in Fig. 3-a the asphericity Δ for different values of *N*_*b*_ as a function of the swimmers area fraction. We find that Δ rapidly decays to zero with increasing *ϕ* especially for large vesicles (*N*_*b*_ = 150), indicating that at high density of swimmers the active vesicle approaches an average circular shape. In contrast, we observe deviation from a circular shape for small vesicles in the dilute regime.

Now we quantify the membrane stretching for *N*_*b*_ = 150 (in this case Δ~0 in the whole *ϕ* range explored). We show in Fig. 3(b) that the gyration radius, *R*_*g*_ increases with *N*_*s*_. This is true for all vesicle sizes (*N*_*b*_ = 50, 100 not shown in figure), indicating that the active particles exert a pressure that stretches the bounding membrane. A simple estimate for the dependence of *R*_*g*_ on swimmer packing fraction can be obtained for a dilute gas of spherical *run-and-tumble* swimmers. In two dimensions the pressure of an ideal active gas of *N*_*s*_ spherical swimmers in an area *A* is the so-called swim pressure^32^,^33^, given by


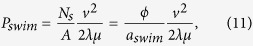


where we have expressed *P*_*swim*_ in terms of the initial packing fraction *ϕ*. In presence of confining structures the pressure in the bulk is strongly affected by the finite size effects^10^,^31^,^32^,^53^.

For example, in the case of one dimensional gas of *run-and-tumble* particles confined in a box of side *L* we can write^53^:


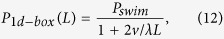


We assume that the internal pressure is responsible of an isotropic deformation of the vesicle from a circle of radius *R*_0_ to a circle of radius *R*_*g*_. In the dilute regime, we assume that Eq. (12) can be recast phenomenologically as


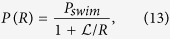


with 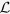 a fitting parameter. A relation between the internal active pressure and the radius *R*_*g*_ in the deformed configuration can be obtained as follows. Since the membrane is composed by elastic springs it will store an elastic energy given by,


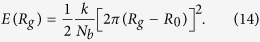


The membrane tension exerts an inward pressure that has to be balanced by the pressure exerted by the active particles, requiring


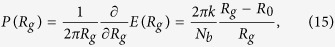


In Fig. 3-(b), the red circles represent the quantity (*R*_*g*_ − *R*_0_)/*R*_*g*_ as a function of the actual area fraction computed as 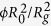. For *N*_*b*_ = 150 the membrane has a nearly circular shape so that (15) holds and (*R*_*g*_ − *R*_0_)/*R*_*g*_ becomes proportional to the average pressure exerted by the swimmers. This is confirmed by plotting on the same graph the swimmers pressure as obtained from simulations and divided by 2*πk*/*N*_*b*_ (black squares). In the dilute regime, Eq. (13) holds, as a result the pressure should scale linearly with the packing fraction, provided the correction term *c*(*R*) and thus *R* does not change significantly with the packing fraction. By fitting the low *ϕ* data in Fig. 3-(b) we obtain *c* = 0.46. Deviations of the pressure from the linear regime, due to the excluded volume effects, are visible at high *ϕ*^10^,^31^,^32^. We can repeat the same procedure for membranes of different contour length and obtain *c* values for different *N*_*b*_. Assuming 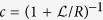 we expect that the quantity *c*/(1 − *c*) should scale linearly with *R* which is approximately proportional to *N*_*b*_ (see inset of Fig. 3-(b)).

**Figure 4:**
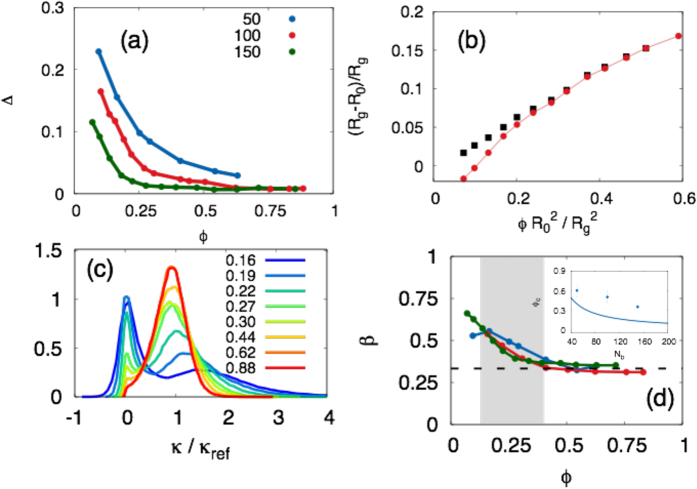
Membrane shape for elongated swimmers. (**a**) Asphericity parameter for *N*_*b*_ = 50 (blue symbols), *N*_*b*_ = 100 (red symbols), and *N*_*b*_ = 150 (green symbols). The lines are a guide to the eye. With increasing *ϕ* the vesicle approaches an average circular shape (Δ ~ 0). (**b**) The red circles are (*R*_*g*_ − *R*_0_)/*R*_*g*_, the red line is a guide to the eye, the black squares represent *PN*_*b*_/2*πk*. The data are plotted as a function of the area fraction computed with respect the circle of radius *R*_*g*_ for *N*_*b*_ = 150. (**c**) The probability distribution of the local curvatures undergoes a crossover from single to double peacked shape by increasing *ϕ* (in figure from 0.16 (blue) to 0.83 (red)). (**d**) To quantify the bimodal character of the distribution we look at the Sarle’s bimodality coefficient *β* as a function of *ϕ* for *N*_*b*_ = 50, 100, 150 (blue, red and green), the black dashed line is *β* for a Gaussian distribution. The grey area represents the estimated *ϕ*_*c*_ range. Inset: *ϕ*_*c*_ obtained from the decoupling between pressure and deformation (blue symbols) the line is the estimate of *ϕ*_*c*_ given by *ϕ*(*N*_*b*_ = *N*_*s*_).

Now we consider elongated swimmers (*p* = 2, aspect ratio *α* = 1/2). In order to evaluate the impact of aligning forces on the membrane shape, we perform numerical simulations of elongated active particles at almost the same area fractions *ϕ* of the previous case. Again, to quantify the deviations of the vesicle from circular shape we display in Fig. 4-a the asphericity Δ. We find that Δ approaches zero with increasing *ϕ*, indicating that at high density of swimmers the active vesicle approaches an average circular shape (see also the snapshot reported in the right panel of Fig. 2). On the contrary for small *ϕ* we observe strong deviation from a circle, as displayed by the four snapshots shown in Fig. 2, left panel.

The radius of gyration increases with *ϕ*, also for elongated swimmers, as one can see in Fig. 4-b. We observe, however, strong deviations from Eq. (15) at low densities where Δ ≠ 0. This is not surprising since the right hand side of Eq. (15) only holds for circular membranes. At high area fractions the vesicle shape becomes more circular (Δ ~ 0) and Eq. (15) applies. We stress that for elongated swimmers, where an aligning torque exists on the boundary, there is not an ideal active gas equation of state like (11)^30^.

### Local shape properties

A useful characterization of the membrane shape is obtained by analyzing the distribution of local curvatures, 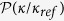, with 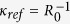 the curvature of the reference circular configuration, shown in Fig. 3-c for spherical swimmers and in Fig. 4-c for elongated swimmers. Low density configurations are generically characterized by pinched spots, where both particle density and curvature are high, separated by straight, low curvature regions that are free from active particles (see the snapshot reported in Fig. 2, left panel).

Let us start by considering 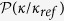 for elongated swimmers, where the asymmetry at low *ϕ* given by Δ(*ϕ*) is more pronounced than for spherical swimmers. The distribution changes from bimodal to unimodal with increasing packing fraction *ϕ*. The bimodal distribution obtained at low *ϕ* corresponds to elongated vesicles. The two peaks correspond to low curvature regions (where *κ* → 0 and the density of active swimmers is very low) and high curvature regions (where swimmers accumulate and *κ* > *κ*_*ref*_), respectively. At large *ϕ* the vesicles are spherical on average (Δ ~ 0) and the distribution of curvature exhibits a single peak. The finite width of the distribution measures the size of fluctuations about the mean shape with 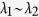.

The bimodal character of the distribution can be quantified using the Sarle’s bimodality coefficient *β* = (*γ*^2^ + 1)/*k*, where *γ* is the skewness and *k* the kurtosis of the distribution. Figure 4-c shows the parameter *β* reported as a function of swimmer density and for three vesicle sizes. Deviations from the 1/3 value, corresponding to a normal distribution, observed at low swimmer density signal the appearance of the bimodality and are associated to elongated vesicle shape. Particles tend to accumulate in small regions, enhancing the local membrane curvature, and leaving large parts of the membrane empty. The empty regions are flat and give a peak at a vanishing value of the local curvature. This results from a positive feedback mechanism by which a local fluctuation of particles density produces a local pressure increase that increases the local curvature on the flexible membrane. Since active particles tend to accumulate at convex boundaries, this local curvature increase drives further accumulation of swimmers.

**Figure 5:**
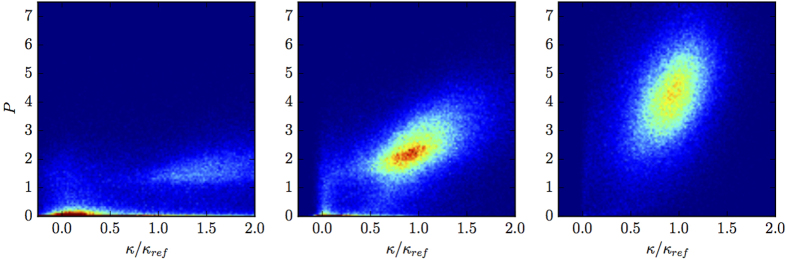
Local curvature. Joint probability distribution of the local curvature of the membrane *κ*/*κ*_*ref*_ (*κ*_*ref*_ is the reference curvature of the circular free membrane) and the local pressure *P* exerted by the swimmers on the membrane. The three panels refer to three different swimmers density, *ϕ* = 0.16 (left), 0.51 (middle) and 0.83 (right). Data correspond to the case of elongated swimmers and a membrane of *N*_*b*_ = 100 beads.

Figure 5 shows the joint probability density 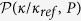. In the low density regime (left panel of Fig. 5), flat regions of the membrane–peak close to (0, 0) in the figure–coexist with highly curved regions–lighter regions close to (1.5, 1.5) in the figure (see also the snapshots reported in Fig. 2). By increasing the number of swimmers inside the vesicle the spot close to the origin disappears and a single broad peak at high *κ*/*κ*_*ref*_ survives corresponding to uniform curvature of the membrane–see the snapshots of Fig. 2, right panel, corresponding to the high particles density.

Similar results are obtained also for spherical swimmers, where the curvature distribution evolves from double to single peaked with increasing area fraction *ϕ*. In this case, however, this transition is sharper and occurs at lower values of *ϕ*, and vesicles display a nearly circular shape in a wider range of area fractions.

The crossover from single to double peaked distribution of the membrane curvature relies on the imbalance of swimmers along the boundaries. A rough estimate of the packing fraction *ϕ*_*c*_ at which the crossover takes place can be obtained by the following argument. A membrane composed by *N*_*b*_ beads of diameter *a* has a length *aN*_*b*_. The minimum number of swimmers of thickness *a* and length 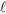 needed to uniformly cover the entire length of the membrane is *N*_*b*_ (we suppose that the swimmers are pushing the membrane and that they are perpendicular to it). The area fraction of swimmers is defined as *ϕ* = *N*_*s*_*a*_*swim*_/*A*_*ref*_, where *A*_*ref*_ = (*N*_*b*_*a*_*swim*_)^2^/4*π* is the area of the reference circular configuration of the free membrane. The critical area fraction of swimmers is then *ϕ*_*c*_ = *ϕ*(*N*_*s*_ = *N*_*b*_) = *pπ*^2^/*N*_*b*_. This corresponds to the minimal swimmers density needed to obtain a uniform distribution of pushing active particles along the membrane. We obtain values of *ϕ*_*c*_ ranging 0.4 to 0.13 in the case of elongated swimmers, and values from 0.2 to 0.07 in the case of spherical swimmers, in agreement with the crossover regions observed in the behavior of *β* (Figs 3-d and 4-d where the grey area represents the *ϕ*_*c*_ range).

A numerical estimate of *ϕ*_*c*_ is obtained as follows. When Δ ≠ 0, Eq. (15) does not hold and the relative displacement (*R*_*g*_ − *R*_0_)/*R*_0_ is not proportional to the average pressure exerted by the active particles. We define *ϕ*_*c*_ as the value of *ϕ* where Eq. (15) begins to hold. In the inset of Fig. 3-d the line is the estimate of *ϕ*_*c*_ given by *ϕ* (*N*_*b*_ = *N*_*s*_) and the symbols are the numerical values (spherical swimmers) obtained looking at the deviation from Eq. (15). The curve reproduces quite well the data. Different is the situation for the elongated swimmers (inset in Fig. 4-d), where the numerical estimate lies above the curve *ϕ*(*N*_*b*_ = *N*_*s*_), i. e., the steric effect is not enough to justify the rise in *ϕ*_*c*_.

### Cell migration

Flexible vesicles do not just fluctuate in shape but, at the same time, perform a random walk under the action of the fluctuating force arising from the combined action of swimmers’ propelling forces. The case of spherical swimmmers is particularly remarkable since it can be worked out analytically. Since swimmers and passive beads have the same size and mobility, the center of mass velocity **V**_*cm*_ is given by


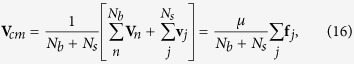


where **V**_*n*_ and **v**_*j*_ are respectively the velocities of a membrane bead and a swimmer. The sum of all interaction forces has to vanish so that only the sum over propelling forces **f**_*j*_ survives in the last term. Therefore the center of mass moves as a body of reduced mobility *μ*/(*N*_*b*_ + *N*_*s*_) under the action of the total propelling force on the swimmers. The corresponding velocity-velocity correlation function is then given by


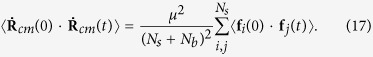


For spherical swimmers, propelling forces only reorient due to tumbles and are therefore uncorrelated so that


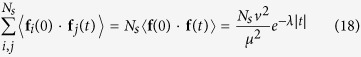


The mean square displacement (MSD) is obtained by a double time integration of (17), with the result


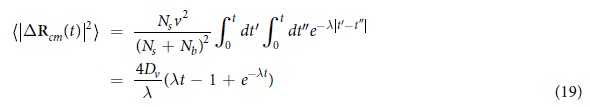


**Figure 6:**
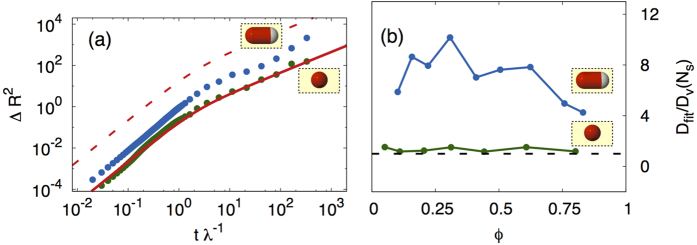
Vesicle motion. (**a**) Mean square displacement of the vesicle center of mass for *ϕ* = 0.16 and *N*_*b*_ = 100. Data correspond to the cases of elongated swimmers (blue symbols) and spherical swimmers (green symbols). The red curve is the theoretical prediction given by Eq. (19). The red dashed curve is the theoretical mean square displacement of a free run-and-tumble particle. (**b**) Diffusion coefficient normalized to the reduced value *D*_*v*_(*N*_*s*_) for elongated (blue symbols) and spherical (green symbols) swimmers as a function of swimmer density *ϕ* (*N*_*b*_ = 100). The parameters are obtained by fitting the data with Eq. (19). The black dashed line is *D*_*fit*_/*D*_*v*_ = 1.

The MSD of the center of mass of the vesicle is then given by the MSD of an individual swimmer, with the single swimmer diffusivity *D* = *v*^2^/2*λ* replaced by the reduced value *D*_*v*_ = *DN*_*s*_/(*N*_*s*_ + *N*_*b*_)^2^. The MSD of a free swimmer^54^ and of a vesicle filled with spherical swimmers are shown in Fig. 6-a together with the formula (19). The case of non spherical swimmers is more complex due to the rotational couplings between propelling forces induced by anisotropic interactions. Still the calculated MSD can be fitted with formula (19) leaving both *D*_*v*_ and *λ* as free fitting parameters. In this case, however, we expect that due to anisotropic interactions, correlations between **f**_*j*_ will arise whose relaxation is not solely driven by the tumbling rate *λ* but can occur on longer time scales. The obtained fitting parameters confirm those expectation giving 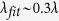.

The fitted diffusion coefficients as a function of particles density are reported in Fig. (6-b) for both spherical and elongated swimmers. As expected, the diffusion coefficient in the spherical case is given by the reduced value *D*_*v*_. In the case of elongated swimmers the vesicle diffusivity is much larger due to a longer persistence of propelling forces arising from locally aligned configurations of swimmers.

## Discussion

Understanding the properties of active matter in confined geometries is of great importance not only for basic science, but also for possible practical applications, for example in micro bio-mechanics, where synthetic autonomous self-propelled objects could be used as drug-delivery agent or for mechanical actuation. Previous studies have focused on the behavior of active particles in the presence of rigid obstacles or confined by stiff boundaries^34^,^35^,^36^,^38^. In this paper we explore the shape changes and spontaneous migration of a flexible vesicle filled with active particles. We find strong fluctuations of the vesicle’s shape, changing from circular to elongated with decreasing number of enclosed particles. The transition between these two regimes is associated with the crossover of the distribution of the local curvatures 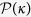 from single-peaked to bimodal. The observed shape deformation is driven by the accumulations of active particles in the high curvature regions, which has been observed also in the case of non interacting Active Brownian particles under strong confinement^38^. Elongated swimmers enhance shape deformations because alignment tends to increase particle accumulation in high curvature regions.

We have recently become aware of a study similar to ours investigating shape fluctuations in 2D flexible vesicles filled with *spherical* Active Brownian particles^42^. Although in this work particles’ trajectories are randomized by rotational diffusion while we use run-and-tumble dynamics, both our work and ref. 42 find similar robust shape fluctuations induced by the active particles. The transition from elongated to circular vesicle shape that we observed by increasing density of enclosed swimmers is found in ref. 42 upon decreasing the particles’ propelling force.

We also show that the filled vesicle effectively behaves like an active object, with exponentially correlated random motion, whose properties are strongly dependent on the shape and density of the self-propelled pushing particles inside. In the case of spherical swimmers we can calculate the diffusion coefficient *D*_*v*_ and the correlation time *τ* of the persistent random walk of the filled vesicle, that can be described in terms of an effective temperature that depends on the number of enclosed swimmers. The migratory properties of the cell are determined entirely by the motility of the active particles.

We additionally examine the behavior of vesicles filled with elongated particles that was not considered in ref. 42. In this case the diffusion coefficient of the whole vesicle is about one order of magnitude greater than that of the spherical case and it is a non-monotonic function of the swimmers density, reaching a maximum value near the critical packing fraction *ϕ*_*c*_ controlling the crossover from single to double peaked distribution of the membrane curvature.

The behavior of vesicles filled with active particles bears some resemblance with the directed migration of Eukaryotic cells, as observed for instance in wound healing assays or in the presence of chemotactic cues. In these situations cells become polarized and perform directed random walks advancing preferentially toward or away from chemical stimuli^44^ or towards regions void of other cells^55^. Our work shows (see Fig. 6b) that vesicle migration is most effective when driven by elongated particles that indeed induce a net polarization of the vesicle, as observed in the chemotactic motion of living cells. It would be interesting to study the effect of chemotaxis on our model by considering a space-varying tumbling rate *λ*(**r**) which depends on an external chemotactic field *c*(**r**).

## Additional Information

**How to cite this article**: Paoluzzi, M. *et al*. Shape and Displacement Fluctuations in Soft Vesicles Filled by Active Particles. *Sci. Rep.* **6**, 34146; doi: 10.1038/srep34146 (2016).
